# An Examination of Public Knowledge of Mild Traumatic Brain Injury

**DOI:** 10.1002/puh2.70075

**Published:** 2025-07-04

**Authors:** Taylor Zurlinden, Gillian Falletta, Kate Schneider, Xanthia Saganis, Anne Sorrell, Anya Savransky, D. Erik Everhart

**Affiliations:** ^1^ 366th Medical Group, Mountain Home Air Force Base Mountain Home Idaho USA; ^2^ Department of Psychology East Carolina University Greenville North Carolina USA

**Keywords:** concussion, health knowledge, public health education, traumatic brain injury

## Abstract

**Introduction:**

Almost three million traumatic brain injuries (TBIs) are reported in the US annually. Concussions, also known as mild TBIs (mTBIs), are the most common and account for approximately 90% of TBIs annually. Research indicates confusion regarding the (1) symptoms, (2) mechanisms of injury, and (3) treatment and recovery associated with mTBI. This study sought to build upon previous research by investigating mTBI knowledge within these three domains.

**Materials and Methods:**

Responses from 529 participants were collected from two groups: college students (*n* = 333) and the general public (*n* = 196). Participants completed a self‐report questionnaire that included true/false items spanning the three domains of mTBI knowledge.

**Results:**

Overall, mTBI knowledge accuracy was 67%, with highest accuracy on items related to mTBI symptoms (78.77%), followed by mechanisms of injury (71.6%) and treatment and recovery (53.9%). College students had significantly higher mTBI knowledge accuracy, as well as higher accuracy on the mTBI symptom and mechanism items. There were no significant differences in knowledge of mTBI treatment and recovery between groups.

**Discussion:**

This study highlights the importance of mTBI education following an mTBI diagnosis. Explanations for these findings considering demographic and individual differences are provided, and clinical implications are discussed.

## Graphical abstract

There is confusion regarding the symptoms, mechanisms of injury, and treatment and recovery associated with mTBI. This study sought to build upon previous research by investigating mTBI knowledge among college students and the general population. Overall, mTBI knowledge accuracy was 67%, which highlights the importance of education following mTBI.

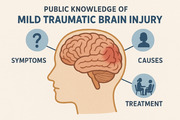

## 1 Introduction

Traumatic brain injury (TBI) is “an alteration in brain function or other evidence of brain pathology caused by an external force” (p. 1; [1]). Severity is determined by the presence and duration of loss of consciousness, posttraumatic amnesia, and initial score on the Glasgow Coma Scale (GCS; [2, 3]). The extent of alterations in language, consciousness, and motor function leads to classification as mild, moderate, or severe. Mild TBIs (mTBIs), also known as concussions [4], are the most common; according to the US Center for Disease Control and Prevention (CDC), mTBIs account for approximately 75% of reported brain injuries [5]. The Diagnostic and Statistical Manual of Mental Disorders (DSM‐5‐TR; 1) classifies mild neurocognitive disorder due to mTBI as lost consciousness for 30‐min or less, posttraumatic amnesia for 1 day or less, altered consciousness for 1 day or less, and a GCS score of 13–15 within 30 min after injury. Neuroimaging scan images must be normal for mTBI classification; abnormal scans indicate “complicated mTBI.”

### 1.1 mTBI Knowledge Accuracy Research

In 1988, Gouvier et al. [6] examined common misconceptions about TBI held by the public and assessed current broad TBI‐related knowledge (i.e., knowledge accuracy) in the public. After surveying over 200 participants, they found that individuals had a knowledge accuracy of 58% and commonly misunderstood the symptoms and sequelae of brain injuries. Specifically, approximately 50% of participants incorrectly responded to items related to unconsciousness, amnesia, and recovery following TBI. This study also examined how people learned about brain injuries; the two most frequently reported sources of information were conversations with medical professionals and watching television talk show hosts (42% for both; 6). Overall, the participant's low knowledge of TBI suggests overestimation of the reliability of non‐medical sources.

A more recent study by Hux et al. [7] found their participants to have a knowledge accuracy rate of 62% and reported that many participants held misconceptions about TBI relating to coma, unconsciousness, memory deficits, and the recovery process. The authors suggested that this may be due to an overreliance on non‐credible sources for TBI information (i.e., visual media), which are often sensationalized or fictionalized. Additionally, a study by Guilmette et al. [8] found their participants to have an overall knowledge accuracy rate of 56%. Interestingly, the results suggested that participants were more knowledgeable about mTBI compared to moderate and severe TBI.

To further explore participant knowledge of mTBI, Block et al. [9] assessed knowledge accuracy of mTBI in a sample of veterans. This study found that veterans were able to better identify true mTBI items but endorsed several items not typical of an mTBI. Notably, having sustained a prior mTBI and receipt of brain injury‐related education were not associated with level of knowledge. Merz et al. [10] explored broad TBI (including mTBI) knowledge accuracy in the public and found a 61% accuracy rate. Interestingly, prior mTBI diagnosis, formal mTBI training, and being an athlete were associated with lower overall TBI knowledge accuracy [10]. Accordingly, more research is needed to assess the areas of mTBI that participants have the most and least accurate knowledge of, as well as the factors (e.g., demographic information, prior mTBI diagnosis, and perceived accuracy of mTBI media portrayals) that are associated with accurate knowledge of mTBI.

### 1.2 Rapidly Changing Understanding of mTBI in the Literature

One factor leading to confusion about mTBI may be the lack of consensus regarding definition; Crowe et al. [11] identified 17 definitions of the term via systematic review. There is also a lack of consensus regarding treatment strategies. For example, Lebrun et al. [12] reported at least 17 different mTBI management guidelines based on clinical practice rather than research, potentially leading to confusion about best practices for providers [13, 14]. Additionally, there is a paucity of research with strong methodology that informs mTBI intervention strategies, which is a barrier to standardized clinical guidelines. A systematic review of effective rehabilitation strategies by Resch et al. [15] analyzed 20 studies published between the years 1987 and 2014 and rated only one as having a “strong quality” based on study design, data collection methods, intervention integrity, lack of confounding variables, and selection bias. One barrier to conducting high‐quality research may be the heterogeneity of mTBIs, as symptoms vary based on several factors such as location of injury and patient age [16], as well as psychosocial factors including mental health concerns (i.e., anxiety, depression, and emotional distress) prior to and post head injury (see de Neeling [17] for a review). Overall, the lack of consistency in the current evidence base and new developments in TBI research may have downstream effects on the public's knowledge of mTBI.

### 1.3 The Present Study

This study sought to replicate and expand on previous research [6, 7, 8, 9, 10] by assessing the content areas of mTBI knowledge. The present study included survey items related to three areas of mTBI knowledge: (1) symptoms, (2) mechanisms of injury, and (3) treatment and recovery. mTBI knowledge accuracy within a college student and general public sample was investigated to examine misconceptions. A convenience sample was used to explore which of the three areas of mTBI knowledge are most and least accurately understood by the public. College students were recruited separately from the general public, as the college students at the university of recruitment receive up to date education about TBI in current coursework. The potential associations between mTBI knowledge accuracy and demographic and individual differences (e.g., previous mTBI history and perceived accuracy or mTBI portrayals) were also explored. This study sought to establish a foundation for understanding gaps in accurate, available mTBI information, enabling future identification of remedies to bridge this gap. Although novel TBI research continues to develop, it is important for the public to have a working knowledge of the symptoms, mechanisms of injury, and treatment and recovery guidelines as this may improve outcomes following head injury. By elucidating the areas of mTBI knowledge participants have the most and least accurate knowledge of, researchers can better inform interventions, healthcare providers, and public awareness campaigns to reduce misconceptions about mTBI in more distinct knowledge areas. Additionally, some items related to knowledge of moderate and/or severe TBI were included; the items selected were added due to the lower rates of correct responses on these items in previous studies [6, 7, 8, 9, 10].

## 2 Materials and Methods

### 2.1 Participants and Procedure

This study was approved by the University Medical Center Internal Review Board (UMCIRB #20‐001814) at East Carolina University. A college student sample (*n* = 333) and general public sample (*n* = 196) were recruited online via email announcements on Listservs, social media posts, and through research recruitment systems (Sona Systems and Amazon Mechanical Turk, or MTurk). Eligibility criteria included being >18 years of age and living in the United States at the time of participation. As compensation for study completion, college participants recruited through Sona Systems received course extra credit, whereas those from the public recruited through Amazon MTurk received $1.80. Participants recruited through social media posts (e.g., Facebook or X, formerly known as Twitter) did not receive compensation. See Table 1 for demographic characteristics of the sample.

**TABLE 1:** Sociodemographic characteristics of participants.

	General public		College students	
	*N*	%	*N*	%
**Gender**				
Man/Male	100	51.0	107	32.1
Woman/Female	95	48.5	225	67.6
Other‐identifying	1	0.5	1	0.3
**Race**				
African American/Black	11	5.6	44	13.2
Alaska Native/Native American	2	1.0	2	0.6
Asian	14	7.1	13	3.9
Multi‐racial	1	0.5	22	6.6
Other	0	0	12	3.6
White	168	85.7	240	72.1
**Highest educational level**				
High school/GED	13	6.6	155	46.5
Some college	26	13.3	168	50.5
2‐year degree	17	8.7	8	2.4
4‐year degree	97	49.5	0	0
Post‐college	43	21.9	1	.3
**Geographical residence**				
Western United States	34	17.3	0	0
Midwestern United States	36	18.4	3	0.9
Southern United States	74	37.8	306	91.9
Northeastern United States	52	26.5	24	7.2
**Occupational status**				
Full‐time	145	74.0	20	6.0
Part‐time	24	12.2	82	24.6
Unemployed‐seeking	3	1.5	23	6.9
Unemployed‐non‐seeking	6	3.1	10	3.0
Retired	6	3.1	2	0.6
Student	10	5.1	195	58.6
Disabled	2	1.0	1	0.3

*Note:* Other identifying includes participants who identify as non‐binary (*n* = 1) and transgender (*n* = 1). No participants had less than a high school education.

### 2.2 Measures

#### 2.2.1 mTBI Knowledge Assessments

All participants completed an online self‐report questionnaire via Qualtrics with true/false items spanning three areas of mTBI knowledge: symptoms, mechanisms, and treatment and recovery. For the items pertaining to symptoms of mTBI, participants were asked to indicate whether the symptoms listed are commonly experienced within 24‐h of sustaining an mTBI by selecting either “true” or “false.” Additionally, a total percentage correct was calculated (i.e., mTBI knowledge accuracy), as well as percentage correct for each of the three areas. Accuracy was calculated by dividing the total number of correct answers by the total number of items answered (i.e., 38) to get the percentage of correct answers. One point was allotted for each correct answer, contributing to the overall score. Studies such as those conducted by Hux et al. [7] and Merz et al. [10] have similarly defined knowledge accuracy in this manner. Items used in the current study were adapted from Gouvier et al. [6] and Merz et al. [10]; several of the symptom items were adapted from Wallace et al. [18]. Additional items were generated on the basis of current literature. Most items included in the survey were related to mTBI; however, some items pertained to moderate or severe TBIs.

Additional survey items were included to assess individual differences that may contribute to mTBI knowledge. Participants were asked to rate the extent to which they identified as an athlete on a scale of 0–100 (0 = not at all, 100 = a lot). Next, participants were asked to report any historical mTBI or TBI diagnosis from a healthcare professional. They also rated their perceived accuracy of mTBI portrayals from television/movies on a scale of 0–100 (0 = not at all accurate, 100 = completely accurate). Finally, participants were asked to rate confidence in their accuracy of mTBI knowledge on a scale of 0–100 (0 = not at all confident, 100 = completely confident).

### 2.3 Validity Items

Several validity checks were embedded throughout the survey. These checks instructed participants to respond to items in a specific way (e.g., “answer ‘yes’ on this item”). Additionally, some of the multiple‐choice items included an “other” response option in which participants could select “other” and use the free textbox to input a response not already included as a multiple‐choice option. A free textbox was also available for participants to provide any additional comments regarding their experiences following head injury. As a result, written responses were reviewed by study personnel to ensure participants included their own, original responses. Answers that were not related to the prompt or study (e.g., famous poems) were considered invalid responses. After failing two embedded validity checks, the participant's data were discarded.

### 2.4 Analyses

All data were analyzed utilizing the Statistical Package for the Social Science (IBM SPSS Statistics, Version 26.0). Correlation analyses were used to assess associations between demographic and individual differences (e.g., identity as an athlete, previous mTBI history, and perceived accuracy of mTBI media portrayals) with total mTBI knowledge accuracy, as well as mTBI symptom accuracy, mechanism accuracy, and treatment and recovery accuracy. Group differences between college students and the general public were examined using analysis of variance (ANOVA). Post hoc multiple linear regression analyses were performed to assess that demographic and individual difference factors significantly predicted mTBI knowledge accuracy.

## 3 Results

### 3.1 mTBI Knowledge Items

Across mTBI knowledge categories, total mTBI knowledge accuracy was 67%. Cronbach's alpha for the 21 symptom items was 0.759 and in the acceptable range. For the six mechanisms of injury and 11 treatment and recovery items, Cronbach's alphas were 0.250 and 0.070, respectively. The low internal consistency of these items is not unexpected given the heterogeneity of mTBIs and TBIs and the broad range of items included as part of these two domains.

Participants had the highest accuracy on mTBI symptom items, with a mean correct response rate of 78.77% (Table 2). Accuracy was higher for identifying true symptoms of mTBI compared to false symptoms, and mTBI symptoms with the highest accuracy rate were sensitivity to light, dizziness, and confusion. False symptoms, including nosebleeds, sharp burning neck pain, jaw pain, and numbness in neck, were frequently identified incorrectly as mTBI symptoms.

**TABLE 2:** Mild traumatic brain injury (mTBI) symptom accuracy.

Question accuracy	
**False symptoms**	**Percent correct**
Numbness in neck	49.2
Sharp burning pain in neck	50.2
Nosebleed	53.2
Jaw pain	58.0
Abnormal sense of smell	60.0
Abnormal sense of taste	66.3
Black eye	68.0
Difficulty breathing	70.6
Chest pain	80.7
**True symptoms**	**Percent correct**
Vomiting	75.2
Sensitivity to sound	79.9
Loss of consciousness	82.8
Memory loss	82.8
Fogginess	83.0
Blurred vision	84.7
Sleep problems	84.5
Nausea	85.0
Balance problems	87.7
Dizziness	88.3
Confusion	88.1
Sensitivity to light	88.4

*Note:* This table reflects the combined accuracy of items for all individuals surveyed.

For items related to the mechanism of injury, participants had a mean correct response rate of 71.6% (Table 3). Most participants accurately agreed that loss of consciousness is not necessary to receive an mTBI diagnosis (90.9%). Conversely, only about one quarter of participants accurately agreed that loss of consciousness is an indicator of mTBI. Additionally, over 85% of participants accurately agreed that a lack of symptoms following a head injury does not rule out an mTBI diagnosis, nor should an athlete continue to play in a game following head injury even if they are asymptomatic.

**TABLE 3:** Mild traumatic brain injury (mTBI) mechanism accuracy.

False items	Percentage correct
You can be knocked unconscious and still not sustain a TBI	26.5
If an athlete sustains a TBI while playing, they can keep playing in the game if they feel fine	86.7
If you can walk and talk after getting hit hard in the head, then you definitely did not sustain a TBI	87.5
In order to be diagnosed with a concussion, you have to be knocked out	90.9

True items	Percentage correct
Falls are the most common cause of TBIs	64.0
You can sustain a TBI without actually being hit directly in the head	74.2

*Note:* This table reflects the combined accuracy of items for all individuals surveyed.

Participants had the lowest accuracy on items regarding treatment and recovery, with a mean correct response rate of 51.33%. This set of items showed the most variability in accuracy rates; see Table 4. Only about 15% of participants accurately *disagreed* with the statement that “people can forget who they are and not recognize others but be normal in every other way.” Additionally, less than half of participants accurately agreed that most people with an mTBI fully recover within 6 months. Most participants accurately agreed that people can become depressed while recovering from an mTBI and that feeling “back to normal” following an mTBI does not necessarily indicate that the recovery process is complete.

**TABLE 4:** Mild traumatic brain injury (mTBI) treatment and recovery accuracy.

False statements	Percent correct
People can forget who they are and not recognize others but be normal in every other way	15.7
Most people with an mTBI will not have a full resolution from their TBI symptoms until 6 months	38.6
You should not let a person who has experienced a TBI go to sleep	41.3
You should put an individual with a TBI in a dark room immediately following a concussion	40.2
When people are knocked unconscious, most wake up shortly with no lasting effects	42.8
How quickly a person recovers depends mainly on how hard they work at recovering	57.0
Even after several weeks in a coma, when people wake up, most recognize and speak to others right away	71.6
Once a person feels “back to normal,” the recovery process is complete	82.8

True statements	Percent correct
It is not best advice to rest and remain inactive for at least a week during recovery	36.9
After an mTBI occurs, brain imaging (CT scan, MRI, x‐ray, etc.) usually does not show visible physical damage	47.4
Sometimes people become depressed during the recovery period	90.4

*Note:* This table reflects the combined accuracy of items for all individuals surveyed.

### 3.2 Factors Related to mTBI Accuracy

The association between demographic and individual differences on mTBI knowledge accuracy was assessed. Results indicated that older age (*r* = −0.178, *p* < 0.001) and higher education level (*r* = −0.262, *p* < 0.001) were associated with lower total mTBI knowledge accuracy. Findings were similar for mTBI symptom accuracy, such that older age (*r* = −0.202, *p* < 0.001) and higher education level (*r* = −0.289, *p* < 0.001) were significantly associated with lower mTBI symptom accuracy. Higher levels of education (*r* = −0.230, *p* < 0.001) and older age (*r* = −0.179, *p* < 0.001) were also significantly associated with lower mTBI mechanism accuracy. Age and education were not significantly associated with mTBI treatment and recovery accuracy.

Regarding individual differences, higher self‐reported athlete identity was significantly associated with lower overall mTBI accuracy (*r* = −0.088, *p* = 0.044) and with history of formal mTBI diagnosis (*r* = 0.177, *p* < 0.001), though these associations were weak. There was no significant association between the previous mTBI diagnosis and mTBI knowledge accuracy (*r* = −0.031, *p* = 0.472). Higher perceived accuracy of TV/movie portrayals of mTBI was associated with lower mTBI accuracy (*r* = −0.286, *p* = <0.001). mTBI knowledge confidence was *not* associated with overall mTBI knowledge accuracy (*r* = −0.030, *p* = 0.490).

Regarding the three components of mTBI accuracy, there was no association between prior mTBI diagnosis and knowledge of mTBI symptoms (*r* = −0.030, *p* = 0.487), mechanism (*r* = −0.050, *p* = 0.252), or treatment and recovery (*r* = 0.010, *p* = 0.812). Higher athlete identity was associated with lower mTBI symptom accuracy (*r* = −0.102, *p* = 0.019), but not mTBI mechanism accuracy (*r* = −0.011, *p* = 0.798) or treatment and recovery accuracy (*r* = −0.024, *p* = 0.585). Additionally, higher perceived accuracy of TV/movie portrayals of mTBI was associated with lower mTBI symptom (*r* = −0.286, *p *< 0.001), mechanism (*r* = −0.232, *p* < 0.001), and treatment and recovery (*r* = −0.152, *p* < 0.001) accuracy. mTBI knowledge confidence was *not* associated with mTBI symptom (*r* = −0.066, *p* = 0.132), mechanism (*r* = 0.048, *p* = 0.269), or treatment and recovery (*r* = 0.028, *p* = 0.517) accuracy.

### 3.3 Differences Between Groups

Compared to the general public, college students had higher overall mTBI knowledge accuracy (*F*(1, 527) = 37.575; *p* < 0.001). College students also had higher symptom accuracy (*F*(1, 527) = 40.315; *p* < 0.001) and mechanism accuracy (*F*(1, 527) = 40.317; *p* < 0.001) compared to the general public. There was no difference between groups regarding mTBI treatment and recovery accuracy (*F*(1, 527) = 0.330; *p* = 0.566). Expectedly, college students had lower mean years of college education completed (*M* = 2.56 years) compared to the general public, who had a mean of 4.67 years of college education completed (*r* = −0.772, *p* < 0.001).

The general public (*M* = 47.44%, SD = 22.751) endorsed higher perceived accuracy of TV/movie portrayals of mTBI compared to college students (*M* = 36.86%, SD = 20.456; *F*(1, 527) = 30.344, *p* = <0.001). There was a difference between groups regarding mTBI knowledge confidence (*F*(1, 527) = 10.080, *p* = 0.002), such that the general public endorsed higher confidence (*M* = 56.68%, SD = 24.882) than college students (*M* = 49.49%, SD = 25.321). There was no difference between groups regarding past mTBI diagnosis (*F*(1, 527) = 0.275, *p* = 0.600) or athlete identity (*F*(1, 527) = 0.519, *p* = 0.471).

Due to differences among groups regarding age, education, and perceived accuracy of TV/movies and associations with mTBI knowledge accuracy, additional post hoc multiple regression analyses were performed. A model, including age, education, and perceived accuracy of TV/movies, predicted total mTBI accuracy (*F*(3, 528) = 24.729, *p* < 0.001, *R* ^2^ = 0.119). Specifically, education (*b* = −0.201, *p *< 0.001) and perceived accuracy of TV/movie portrayals of mTBI (*b* = −0.238, *p* < 0.001) emerged as predictors of mTBI knowledge accuracy, whereas age (*b* = −0.015, *p* = 0.764) did not. College students with lower perceived accuracy of TV/movie portrayals of mTBI displayed higher mTBI knowledge accuracy.

## 4 Discussion

### 4.1 mTBI Knowledge Items

Although millions of mTBIs occur annually [19], evidence suggests that many people have an incomplete understanding of an mTBI diagnosis. Gaps in mTBI knowledge are noted [6, 7, 8, 9, 10], and this study explored which areas of mTBI information (i.e., symptoms, mechanisms, and treatment and recovery) participants have the most and least accurate knowledge of.

Total mTBI accuracy was 67%, highlighting gaps and variability in knowledge. Higher accuracy was observed on items related to mTBI symptoms (78.77%), followed by mTBI mechanisms (71.6%), and mTBI treatment and recovery (53.9%). Regarding mTBI symptoms, participants were asked to indicate whether the symptoms listed are common symptoms experienced within 24‐h of sustaining a concussion/mTBI. Interestingly, participants responded more accurately to items related to true mTBI symptoms (i.e., light sensitivity, dizziness, and confusion) compared to false symptoms (i.e., neck and jaw pain and numbness in neck). These findings suggest misattribution of symptoms of other injuries (broken bones, nerve damage, etc.) to mTBI.

Participant knowledge of mTBI mechanisms of injury was assessed. Although most participants correctly identified that you do not need to lose consciousness to receive an mTBI diagnosis (90.9%), only about one quarter of participants recognized that loss of consciousness following a blow to the head categorically indicates diagnosis of mTBI or TBI. This finding is concerning as nearly 75% of participants may not recognize that a loss of consciousness is cause for concern.

However, over 85% of participants correctly indicated that a lack of symptoms following a head injury does not necessarily mean that an mTBI has not been sustained. Similarly, a majority of participants were aware that athletes who sustain an mTBI during a game should discontinue playing even if they are asymptomatic. This may be due to the increase in mTBI and TBI safety and return‐to‐play guidelines implemented in popular sports such as football [20, 21, 22, 23].

The lowest rates of accuracy were related to mTBI treatment and recovery (53.9%). Best practice for treating mTBI in the medical community has changed, and delays in dissemination of new information may contribute. This may exacerbate diminished consensus among professionals about the definition and classification of mTBI. Additionally, mTBI treatment and recovery are least understood in the literature as novel research continues to develop, so it is unsurprising that this area is least understood amongst the public as well. Consistent with results from Bradford [24], participants in this study overestimated the typical recovery period for an mTBI (i.e., over 6 months). Research suggests that most patients who sustain an mTBI will recover within a week to 3 months [25], whereas a 6‐month recovery time is more common for moderate TBIs [26]. Longer recovery times associated with more serious injuries may be more salient. Recovery expectations can alter the recovery trajectory and may generate nocebo effects [27].

Common “myths” about mTBI treatments dispelled within the medical community remain present within the lay population. For example, only 40% of participants identified that people who have sustained an mTBI or TBI *can* go to sleep and do *not* have to be put in a dark room following injury. Yet, the Brain Injury Association of America confirms that a restful sleep is essential for recovery [28]. Limited activity and sleep 24‐ to 48‐h after an mTBI are recommended and can mitigate fatigue during the recovery process and restore impaired function to the brain [29]. Evidence suggests that it is harmful to prevent sleep after an mTBI, as getting physical and cognitive rest early on in recovery is predictive of better outcomes [30]. Additionally, although rest and sleep are important parts of mTBI recovery, it is not necessary to put an individual who has sustained an mTBI in a dark room or avoid all sensory stimuli (e.g., reading and social interaction) [30]. It is recommended that providers consider a graduated return to activities [13], where individuals can gradually resume typical physical and cognitive activities as long as symptoms remain stable [14]. Some studies suggest that engaging in gradual and light physical activity following mTBI may decrease recovery time [31, 32]. Despite this research, only 36.9% of the current sample identified that it is *not* best advice to rest and remain inactive for at least a week during recovery. However, most participants were aware that just because a person feels “back to normal” following an mTBI does not indicate that the recovery process is complete; this finding was consistent with that of Merz et al. [10]. Thus, it seems that the public is aware that the recovery process following mTBI takes time, but there is confusion regarding the specific actions needed to best recover from an mTBI.

A majority of the sample (90.4%) correctly indicated that people may become depressed while recovering from an mTBI. This percentage of correct responses is consistent with, and higher than, the findings of Block et al. [9], which found that 84.7% of veterans and 68.8% of friends/family members of veterans accurately endorsed that feeling sad or depressed is a symptom following mTBI. Individuals who sustained an mTBI are three times more likely to experience depression compared to individuals without mTBI history, and this risk remains years after the mTBI occurred [33]. Depression may exacerbate symptoms, slow recovery, and decrease quality of life [33, 34], so continued increase in public awareness of depression symptoms following mTBI is an important step in improving treatment outcomes. This increase in awareness of depression symptoms following TBI in the public may be due to the influx in research studies on this topic [35] and organizations dedicated to brain injury awareness, including the CDC “Heads Up” website (https://www.cdc.gov/headsup/index.html), actively educating and providing resources to the public.

#### 4.1.1 Factors Related to mTBI Accuracy and Differences Between Groups

The sample contained college students and members of the general public, and there were notable group differences. Individuals from the general public reported completing higher education levels than college students. Our results showed that college students (with lower education levels as they were still in school) had higher mTBI knowledge accuracy. One might expect that higher education would be associated with higher knowledge accuracy, but our results showed the opposite; college students with lower education levels (as they were still in school) had higher mTBI knowledge accuracy. This may be explained by the proximity of their educational experiences. Current students may be more likely to learn about mTBI through coursework, sports participation, and so on, whereas individuals in the general public may be more likely to obtain their information from popular media, and if they had learned about mTBI in school, this knowledge may have degraded over time or become outdated.

Additionally, some items related to knowledge of moderate and/or severe TBI were included due to the lower rates of correct responses on these items in previous studies [6, 7, 8, 9, 10], which may be related to media portrayals of TBI [10]. Participants in the general public endorsed higher perceived accuracy of TV/movies, which is consistent with overall lower mTBI knowledge accuracy. The general public may be more likely to obtain information through TV/movies; this, coupled with increased belief in the credibility of mTBI media portrayals, may contribute to misconceptions about mTBI. For example, the concept that people can forget who they are and not recognize others but be otherwise normal is a well‐known plot in popular media (e.g., *50 First Dates* and *The Vow*) and may explain why most individuals in this sample are misinformed about memory deficits following a more severe TBI [13]. In‐line with this evidence, only 15% of the participants in this study correctly answered that people *cannot* forget who they are and *not* recognize others but be normal in every other way.

Last, identifying as an athlete was significantly associated with lower mTBI knowledge accuracy. It is known that athletes have a higher risk of sustaining an mTBI, especially if they participate in contact sports [36]. This was exemplified in the current study, as identifying as an athlete was significantly associated with having received a formal mTBI diagnosis in the past. Specifically, higher self‐reported athlete identity was significantly associated with lower mTBI symptom knowledge. This indicates that nonprofessional athletes may not recognize the symptoms of mTBI and, therefore, may not receive appropriate medical care. This notion is corroborated by Fedor and Gunstad [37] who found that student athletes endorsed significantly more “distractor symptoms” (i.e., symptoms not consistent with an mTBI) than non‐athlete controls and thus may have an incomplete understanding of mTBI symptomatology.

#### 4.1.2 Clinical Implications

Patient–provider interactions are an important source of mTBI and TBI knowledge for the general public. Information related to TBI treatment and recovery has changed over the past several decades, emphasizing the necessity of healthcare professionals to provide up‐to‐date information to their patients. In response to the increase in misinformation related to the novel coronavirus—2019, the US Surgeon General urged healthcare providers to provide accurate health information to the public [38]. Strategies listed included proactively engaging in discussions, using technology/social media, and partnering with community groups and organizations. Evidenced by the findings of the present study, many individuals have an incomplete understanding of the current guidelines and recommendations for recovery following an mTBI, highlighting the importance of accurate information from clinicians. The CDC “Heads Up” website (https://www.cdc.gov/headsup/index.html) provides resources for healthcare providers to provide patients, including pre‐made discharge instructions and symptom‐based recovery tips. Healthcare providers may be able to employ these strategies to help dispel misinformation in the public and improve the public's understanding of mTBI.

### 4.2 Strengths and Limitations

This study utilized items from various studies completed over a 30‐year period. This allowed for the assessment of a comprehensive set of facts related to mTBI. Additionally, although this study was comprised of participants predominately from the southeastern United States, there was some geographic variability among participants. The demographic background of this study shows some inclusion of historically underrepresented demographic groups. In our study, the ethnic background of the sample closely mirrors most recent US Census data (https://www.census.gov/data.html); this allows for increased generalizability of the results.

The primary limitation is sole reliance on self‐report data. Though care was taken in item creation (i.e., using items previously validated in research studies and clarifying language that may be outdated or unclear), in some cases, participants may have misunderstood items, misreported information, or engaged in biased or dishonest (i.e., looking up answers) responding, increasing variability of the data. Additionally, for the items pertaining to symptoms of mTBI, participants were asked to indicate whether the symptoms listed are common symptoms experienced within 24‐h of sustaining a concussion/mTBI by selecting either “true” or “false.” The presentation of these items could have impacted participant responses as several of the symptoms could co‐occur with that of an mTBI if other bodily injuries have been sustained as well. Although every effort was made to reduce misunderstanding of items, the reliance on self‐report data via an online study platform could increase the likelihood of this occurring and should be noted.

### 4.3 Future Directions

Future studies may explore methods for providing mTBI information, such as handouts, videos, follow‐up appointments, or individualized training. It would also be beneficial to investigate methods of correcting misinformation about mTBI, as well as providing novel TBI information to the public considering a changing research landscape. Future studies may focus on mTBI knowledge of individuals pre‐ and post‐educational training and follow‐up surveys. Educational interventions could be constructed based on the current literature regarding health misinformation.

## Author Contributions

**Taylor Zurlinden**: conceptualization, investigation, methodology, formal analysis, data curation, writing – original draft, writing – review and editing, project administration. **Gillian Falletta**: conceptualization, writing – review and editing, formal analysis, methodology. **Kate Schneider**: writing – review and editing, formal analysis, conceptualization. **Xanthia Saganis**: conceptualization, writing – review and editing, formal analysis. **Anne Sorrell**: conceptualization, writing – review and editing, formal analysis. **Anya Savransky**: writing – review and editing, formal analysis. **D. Erik Everhart**: conceptualization, investigation, writing – original draft, writing – review and editing, methodology, formal analysis, project administration, supervision.

## Ethics Statement

This research adheres to the policies of the journal. This research was approved by the University Medical Center Internal Review Board (UMCIRB) at East Carolina University. Participants provided informed consent for participation in the study.

## Conflicts of Interest

The authors declare no conflicts of interest.

## Supporting information




**Supporting File 1:** Survey Items 4–26.pdf

## Data Availability

The data that support the findings of this study are available from the corresponding author upon reasonable request.
