# Supplementary material for: An Examination of Public Knowledge of Mild Traumatic Brain Injury
Source: Public Health Chall. 2025 Jul 4;4(3):e70075. doi: 10.1002/puh2.70075 (PMC12231205; doi:10.1002/puh2.70075)
Supplement: Supplementary file 1 — Supporting File 1: Survey Items 4–26.pdf [file PUH2-4-e70075-s001.pdf]

Dissertation: Concussion Knowledge Survey

iQ Score: Fair | Published

Demographics   Block Options

Q1 Age

[ ]

Q2 Gender

- ○ Male
- ○ Female
- ○ Transgender Male
- ○ Transgender Female
- ○ Non-binary/non-conforming
- ○ Other
- ○ Prefer not to answer

Q7 Education Level

- ○ less than high school
- ○ High school graduate/GED
- ○ Some college
- ○ 2 year degree
- ○ 4 year degree
- ○ Master's
- ○ Doctorate

Q6 Ethnicity

- ○ White
- ○ Black or African American
- ○ American Indian or Alaska Native
- ○ Asian
- ○ Native Hawaiian or Pacific Islander
- ○ Other

Q53

Geographic Location

- Northeast
- Midwest
- South
- West

Q54

Referral Source

- SONA/ECU Experimentrak
- Amazon MTurk

Q5

Highest level of athletic participation

- no participation
- recreational
- high school
- college (NOT intramural leagues)
- semi-professional
- professional

Q4

iQ

How much do you identify as:

	A little				A moderate amount					A lot
	0	10	20	30	40	50	60	70	80	90	100
An athlete										

Q3

Which category do you fall into

- college student
- trainee/clinician
- Other

Add Block

Single TBI Question

Block Options

Q9

Throughout this study you will see the terms "concussion" and "mild TBI." A concussion and mild traumatic brain injury (TBI) refer to the same thing, and will be used interchangeably throughout the study.

Q17

Have you ever **intentionally NOT** reported a suspected TBI/Concussion?

- Yes
- No

Display This Question:
If Have you ever intentionally NOT reported a suspected TBI/Concussion? Yes Is Selected

Q18

What was your reason for not reporting it?

- ○ did not think it was serious
- ○ wanted to continue my activity
- ○ fear of consequences
- ○ other

Q10

Even if you have never been diagnosed with a concussion do you think you may have experienced one?

- ○ Yes
- ○ No

Q8

Have you ever been formally diagnosed with a concussion

- ○ Yes
- ○ No

Condition: No Is Selected. Skip To: End of Block.

Q11

Approximately how many TBIs (concussions) have you had?

- ○ 1
- ○ 2
- ○ 3-4
- ○ 5-6
- ○ 7+

Q12

For these next questions think about *your most recent* concussion/TBI

Q13

How did the TBI occur

- ○ motor vehicle accident
- ○ sports
- ○ fall
- ○ other

Q14

Who made the diagnosis of a concussion

- ○ Athletic Trainer
- ○ Emergency Room
- ○ Paramedic/EMTs
- ○ Other physician
- ○ Other

Q15

Did you lose consciousness

- Yes
- No

Q16

Did you feel dazed and confused?

- Yes
- No

Add Block

BAT-LQ

Block Options

Q23

For the questions below, please only select "yes" if you experienced a blow to the head *as the result* of the event. If you have been in a car crash, but did not hit your head as a result of the crash, then you would select "no" for question 1.

Additionally, only select "yes" for loss of consciousness if you lost consciousness as a *direct result* of your head injury. For example, If you fainted and *then* hit your head you would select "yes" for the first column on question 9, but you would not select "yes" for the second column because you lost consciousness *before* you hit your head.

- I understand

Q20

Have you ever experienced a blow to the head?
Listed below are situations in which you may have experienced a blow to the head. For each event listed, please click yes or no to indicate if you have experienced it. If yes, did you lose consciousness and/or were you dazed or confused as a result?

	Did you expereince this event		If you experienced this event: Did you lose consciousness			If you experienced th Were you dazed confused		
	Yes	No	Yes	No	Unsure	Yes	No	Ur
1. In a car crash	○	○	○	○	○	○	○	
2. In a motorcycle crash	○	○	○	○	○	○	○	
3. In an all-terrain or other type of vehicle crash	○	○	○	○	○	○	○	
4. As a pedestrian hit by a vehicle	○	○	○	○	○	○	○	
5. Being hit by a falling object	○	○	○	○	○	○	○	
6. Being hit by equipment	○	○	○	○	○	○	○	
7. Falling down stairs	○	○	○	○	○	○	○	
8. Falling from a high place	○	○	○	○	○	○	○	
9. During a fainting spell	○	○	○	○	○	○	○	
10. During a drug or alcohol blackout	○	○	○	○	○	○	○	
11. While biking	○	○	○	○	○	○	○	
12. While roller blading or skateboarding	○	○	○	○	○	○	○	
13. While horseback riding	○	○	○	○	○	○	○	
14. Click yes for all 3 columns	○	○	○	○	○	○	○	
15. While skiing or snowboarding	○	○	○	○	○	○	○	
16. While skydiving	○	○	○	○	○	○	○	
17. While participating in other sports (ex. football, hockey, baseball, basketball, soccer, lacrosse, boxing, wrestling, martial arts, etc.	○	○	○	○	○	○	○	
18. While on the playground	○	○	○	○	○	○	○	
19. While diving into water	○	○	○	○	○	○	○	
20. Being physically abused	○	○	○	○	○	○	○	
21. While being assaulted or mugged	○	○	○	○	○	○	○	
22. During military or other training exercise	○	○	○	○	○	○	○	
23. During combat	○	○	○	○	○	○	○	
24. Other	○	○	○	○	○	○	○	

Q22

In this area, please record any comments about your responses. **Precede your comments with the number item you are describing.**

Add Block

TBI Knowledge Source

Block Options

Q24

What percentage of your TBI/concussion knowledge came from the following sources (total must equal 100)

print media (newspapers/magazines/books)	0
television/movies	0
social media	0
personal experience (self or close acquaintance)	0
formal TBI training/programming	0
professional training/research	0
websites	0
other	0
Total	0

Q25

How accurate do you believe TV/movie portrayals of individuals with TBIs are?

Not accurate at all — somewhat accurate — completely accurate

0 10 20 30 40 50 60 70 80 90 100

Accuracy

Q26

Have you actively sought out more information about TBIs?

- ○ Yes - a lot
- ○ Yes - some
- ○ Yes - a little
- ○ No

Add Block

CTE Knowledge

Block Options

Q28

These next questions will ask about your knowledge of Chronic Traumatic Encephalopathy also known as CTE

Q27

What percentage of your CTE knowledge came from the following sources (total must equal 100)

print media (newspapers/magazines/books)	0
television/movies	0
social media	0
personal experience (self or close acquaintance)	0
professional athletes	0
medical journals	0
websites	0
other	0
Total	0

Q28

How comfortable would you be allowing your [hypothetical] to play a high contact sport (football, hockey, soccer, etc.) based *only* on your understanding of CTE?

	Not at all comfortable	somewhat comfortable	completely comfortable

	0	10	20	30	40	50	60	70	80	90	100
Comfort											

Q29

Have you actively sought out more information about TBIs?

- ○ Yes - a lot
- ○ Yes - some
- ○ Yes - a little
- ○ No

Add Block

TBI symptoms

Block Options

Q29

True or False: The symptoms below are common symptoms experienced within 24 hours of sustaining a concussion/mild TBI

	True	False
Chest Pain	○	○
Abnormal sense of smell	○	○
Nausea	○	○
Sleep problems	○	○
Sensitivity to light	○	○
Abnormal sense of taste	○	○
Balance problems	○	○
Sharp burning pain in neck	○	○
Vomiting	○	○
Jaw pain	○	○
Memory loss	○	○
Fogginess	○	○
Sensitivity to sound	○	○
Difficulty breathing	○	○
Dizziness	○	○
Confusion	○	○
Numbness in neck	○	○
Black eye	○	○
Blurred Vision	○	○
Nosebleed	○	○
Loss of consciousness	○	○

Add Block

TBI mechanism

Block Options

Q30

You can be knocked unconscious and still not sustain a TBI

- True
- False

Q31

You can sustain a TBI without actually being hit directly in the head

- True
- False

Q32

If you can walk and talk after getting hit hard in the head, then you definitely did not sustain a TBI

- True
- False

Q33

In order to be diagnosed with a concussion you have to be knocked out

- True
- False

Q34

Falls are the most common cause of TBIs

- True
- False

Q35

If an athlete sustains a TBI while playing, they can keep playing in the game if they feel fine

- True
- False

Add Block

Treatment/Recovery

Block Options

Q36

You should put an individual with a TBI in a dark room immediately following a concussion.

- True
- False

Q37

It is not best advice to rest and remain completely inactive for at least a week during recovery.

- True
- False

Q38

You should not let a person who has experienced a TBI go to sleep

- True
- False

Q39

After a mild TBI (concussion), occurs, brain imaging (CAT scan, MRI, X-ray, etc.) usually does not show visible physical damage.

- True
- Fase

Q40

Even after several weeks in a coma, when people wake up, most recognize and speak to others right away.

- True
- False

Q41

Most people with a mild TBI will not have a full resolution from their TBI symptoms until 6 months

- True
- False

Q42

When people are knocked unconscious, most wake up shortly with no lasting effects.

- True
- False

Q43

People can forget who they are and not recognize others but be normal in every other way.

- True
- False

Q44

Sometimes people become depressed during the recovery period

- True
- False

Q45

How quickly a person recovers depends mainly on how hard they work at recovering

- True
- False

Q46

Once a person feels “back to normal,” the recovery process is complete.

- True
- False

Add Block

Block 8

Block Options

Q47

CTE can only be diagnosed after death

- True
- False

Q48

CTE can be caused by a single event/injury

- True
- False

Q49

If someone’s mood changes after a hit to the head, it is because they have CTE

- True
- False

Q50

We do not know exactly what causes CTE

- True
- False

Q51

You can develop CTE from sports even if you never have a head injury

- True
- False

Q52

CTE cannot be treated at this time

- True
- False

Add Block

End of Survey

Survey Termination Options...
